# Automated Imaging Differentiation for Parkinsonism

**DOI:** 10.1001/jamaneurol.2025.0112

**Published:** 2025-03-17

**Authors:** David E. Vaillancourt, Angelos Barmpoutis, Samuel S. Wu, Jesse C. DeSimone, Marissa Schauder, Robin Chen, Todd B. Parrish, Wei-en Wang, Eric Molho, John C. Morgan, David K. Simon, Burton L. Scott, Liana S. Rosenthal, Stephen N. Gomperts, Rizwan S. Akhtar, David Grimes, Sol De Jesus, Natividad Stover, Ece Bayram, Adolfo Ramirez-Zamora, Stefan Prokop, Ruogu Fang, John T. Slevin, Prabesh Kanel, Nicolaas I. Bohnen, Paul Tuite, Stephen Aradi, Antonio P. Strafella, Mustafa S. Siddiqui, Albert A. Davis, Xuemei Huang, Jill L. Ostrem, Hubert Fernandez, Irene Litvan, Robert A. Hauser, Alexander Pantelyat, Nikolaus R. McFarland, Tao Xie, Michael S. Okun, and the AIDP Study Group, Alicia Leader, Áine Russell, Hannah Babcock, Karen White-Tong, Jun Hua, Anna E. Goodheart, Erin Colleen Peterec, Cynthia Poon, Max B. Galarce, Tanya Thompson, Autumn M. Collier, Candace Cromer, Natt Putra, Reilly Costello, Eda Yilmaz, Crystal Mercado, Tomas Mercado, Amanda Fessenden, Renee Wagner, C. Chauncey Spears, Jacqueline L. Caswell, Marina Bryants, Kristyn Kuzianik, Youshra Ahmed, Nathaniel Bendahan, Joy O. Njoku, Amy Stiebel, Hengameh Zahed, Sarah S. Wang, Phuong T. Hoang, Joseph Seemiller, Guangwei Du

**Affiliations:** 1 Department of Applied Physiology and Kinesiology, University of Florida, Gainesville; 2 Norman Fixel Institute for Neurological Diseases, University of Florida, Gainesville; 3 Department of Neurology, University of Florida, Gainesville; 4 J Crayton Pruitt Family Department of Biomedical Engineering, University of Florida, Gainesville; 5 Digital Worlds Institute, College of the Arts, University of Florida, Gainesville; 6 Department of Biostatistics, University of Florida, Gainesville; 7 Department of Radiology, Northwestern University, Chicago, Illinois; 8 Department of Biomedical Engineering, Northwestern University, Evanston, Illinois; 9 Parkinson’s Disease and Movement Disorders Center, Albany Medical Center, Albany, New York; 10 Department of Neurology, Medical College of Georgia at Augusta University, Augusta; 11 Department of Neurology, Harvard Medical School, Boston, Massachusetts; 12 Beth Israel Deaconess Medical Center, Boston, Massachusetts; 13 Department of Neurology, Duke University Medical Center, Durham, North Carolina; 14 Department of Neurology, Johns Hopkins University School of Medicine, Baltimore, Maryland; 15 Massachusetts General Hospital, Boston; 16 Ken and Ruth Davee Department of Neurology, Parkinson’s Disease and Movement Disorders Center, Northwestern University Feinberg School of Medicine, Chicago, Illinois; 17 Department of Medicine, University of Ottawa, Ottawa, Ontario, Canada; 18 The Ottawa Hospital Research Institute, Ottawa, Ontario, Canada; 19 Department of Neurology, College of Medicine, Pennsylvania State University, Hershey; 20 Department of Neurology, University of Alabama at Birmingham, Birmingham; 21 Department of Neurosciences, University of California, San Diego; 22 Department of Pathology, Immunology, and Laboratory Science, University of Florida, Gainesville; 23 Center for Cognitive Aging and Memory Translational Research Institute, University of Florida, Gainesville; 24 Department of Neurology, University of Kentucky, Lexington; 25 Department of Radiology, University of Michigan, Ann Arbor; 26 Morris K. Udall Center of Excellence for Parkinson’s Disease Research, University of Michigan, Ann Arbor; 27 Department of Neurology, University of Michigan, Ann Arbor; 28 Department of Neurology, University of Minnesota, Minneapolis; 29 Department of Neurology, University of South Florida, Tampa; 30 Brain Health Imaging Centre, Centre for Addiction and Mental Health, Toronto, Ontario, Canada; 31 Krembil Brain Institute, University Health Network, Toronto, Ontario, Canada; 32 Department of Neurology, Wake Forest School of Medicine, Winston-Salem, North Carolina; 33 Department of Neurology, Washington University School of Medicine in St Louis, St Louis, Missouri; 34 Department of Neurology, Weill Institute for Neurosciences, University of California, San Francisco; 35 Center for Neurological Restoration, Neurological Institute, Cleveland Clinic, Cleveland, Ohio; 36 Department of Neurology, University of Chicago, Chicago, Illinois; 37 F.M. Kirby Research Center for Functional Brain Imaging, Kennedy Krieger Institute, Johns Hopkins University School of Medicine, Baltimore, Maryland; 38 Russell H. Morgan Department of Radiology and Radiological Science, Johns Hopkins University School of Medicine, Baltimore, Maryland; 39 Department of Neurology, Stanford Movement Disorders Center, Stanford University, Palo Alto, California

## Abstract

**Importance:**

Magnetic resonance imaging (MRI) paired with appropriate disease-specific machine learning holds promise for the clinical differentiation of Parkinson disease (PD), multiple system atrophy (MSA) parkinsonian variant, and progressive supranuclear palsy (PSP). A prospective study is needed to test whether the approach meets primary end points to be considered in a diagnostic workup.

**Objective:**

To assess the discriminative performance of Automated Imaging Differentiation for Parkinsonism (AIDP) using 3-T diffusion MRI and support vector machine (SVM) learning.

**Design, Setting, and Participants:**

This was a prospective, multicenter cohort study conducted from July 2021 to January 2024 across 21 Parkinson Study Group sites (US/Canada). Included were patients with PD, MSA, and PSP with established criteria and unanimous agreement in the clinical diagnosis among 3 independent, blinded neurologists who specialize in movement disorders. Patients were assigned to a training set or an independent testing set.

**Exposure:**

MRI.

**Main Outcomes and Measures:**

Area under the receiver operating characteristic curve (AUROC) in the testing set for primary model end points of PD vs atypical parkinsonism, MSA vs PSP, PD vs MSA, and PD vs PSP. AIDP was also paired with antemortem MRI to test against postmortem neuropathology in a subset of autopsy cases.

**Results:**

A total of 316 patients were screened and 249 patients (mean [SD] age, 67.8 [7.7] years; 155 male [62.2%]) met inclusion criteria. Of these patients, 99 had PD, 53 had MSA, and 97 had PSP. A retrospective cohort of 396 patients (mean [SD] age, 65.8 [8.9] years; 234 male [59.1%]) was also included. Of these patients, 211 had PD, 98 had MSA, and 87 had PSP. Patients were assigned to the training set (78%; 104 prospective, 396 retrospective) or independent testing set, which included 145 (22%; 60 PD, 27 MSA, 58 PSP) prospective patients (mean age, 67.4 [SD 7.7] years; 95 male [65.5%]). The model was robust in differentiating PD vs atypical parkinsonism (AUROC, 0.96; 95% CI, 0.93-0.99; positive predictive value [PPV], 0.91; negative predictive value [NPV], 0.83), MSA vs PSP (AUROC, 0.98; 95% CI, 0.96-1.00; PPV, 0.98; NPV, 0.81), PD vs MSA (AUROC, 0.98; 95% CI, 0.96-1.00; PPV, 0.97; NPV, 0.97), and PD vs PSP (AUROC, 0.98; 95% CI, 0.96-1.00; PPV, 0.92; NPV, 0.98). AIDP predictions were confirmed neuropathologically in 46 of 49 brains (93.9%).

**Conclusions and Relevance:**

This prospective multicenter cohort study of AIDP met its primary end points. Results suggest using AIDP in the diagnostic workup for common parkinsonian syndromes.

## Key Points

**Question:**

Does 3-T magnetic resonance imaging paired with machine learning meet primary end points for differentiating Parkinson disease (PD), multiple system atrophy (MSA) parkinsonian variant, and progressive supranuclear palsy (PSP)?

**Findings:**

The multicenter Automated Imaging Differentiation of Parkinsonism cohort study of 249 patients and a retrospective cohort of 396 patients showed excellent discrimination of PD vs atypical parkinsonism, MSA vs PSP, PD vs MSA, and PD vs PSP. AIDP machine learning predicted postmortem neuropathology in 93.8% of autopsy cases.

**Meaning:**

Results of this study suggest the use of Automated Imaging Differentiation of Parkinsonism in the diagnostic workup for common neurodegenerative forms of parkinsonism.

## Teaser

This cohort study investigates if 3-T magnetic resonance imaging paired with machine learning meets primary end points for differentiating Parkinson disease, multiple system atrophy parkinsonian variant, and progressive supranuclear palsy.

## Introduction

The application of an imaging-based approach for the diagnosis and differentiation of Parkinson disease (PD), multiple system atrophy (MSA) parkinsonian variant, and progressive supranuclear palsy (PSP) has proven challenging.^1,2,3^ Ideally, an applied methodology would seamlessly integrate into an existing diagnostic workflow.^4^ Analytical approaches that use the standard acquisition of brain magnetic resonance imaging (MRI) scans, commonly obtained in the diagnostic workup of degenerative brain diseases, would be an attractive option. MRI can be rapidly collected, does not require a radiotracer, and its analysis can be completely automated.

The US Food and Drug Administration approved the use of dopamine transporter imaging (DaT SPECT) in 2011 to differentiate parkinsonism from essential tremor. However, the use of a radioactive drug, high associated costs, and a multihour time commitment all limit widespread clinical use. Additionally, this test cannot reliably differentiate between neurodegenerative forms of parkinsonism that involve dopaminergic deficiency, including MSA and PSP.^5,6^ In contrast, recent data indicate that the differentiation of PD, MSA, and PSP is possible when diffusion-weighted MRI is paired with appropriate disease-specific machine learning algorithms.^7^

Based on the positive results from a retrospective study,^7^ a less than 10-minute 3-T diffusion MRI free water (FW)–based imaging sequence—along with automated image processing and support vector machine (SVM) learning—has the potential to improve diagnostic classification of PD and atypical parkinsonism. The field lacks a prospective multicenter study to test if the Automated Imaging Differentiation for Parkinsonism (AIDP) approach meets the primary end points to be considered in a diagnostic workup. To this end, we prospectively evaluated the discriminative performance of AIDP across 4 primary end points, including PD vs atypical parkinsonism, MSA vs PSP, PD vs MSA, and PD vs PSP. The reference ground truth was the clinical diagnosis confirmed by 3 independent, blinded neurologists who specialize in movement disorders. Additionally, we paired AIDP with antemortem MRI to test against postmortem confirmed neuropathology in a subset of cases.

## Methods

This was a prospective cohort study conducted across 21 Parkinson Study Group centers in the US and Canada from July 2021 to January 2024 (eMethods in Supplement 1). Written informed consent was obtained from all participants. Study procedures were conducted per the ethical standards approved and monitored by the University of Florida institutional review board. The study was overseen by a steering committee of neurologists specializing in movement disorders (N.R.M., M.S.O., I.L., H.F., T.X., A.P., R.A.H.). We followed the Strengthening the Reporting of Observational Studies in Epidemiology (STROBE) reporting guidelines.

### Participants

Patients aged 40 to 80 years with PD, MSA parkinsonian variant, and PSP were eligible. The prospective cohort included participants who self-identified with the following races and ethnicities: Asian, Black, Hispanic, non-Hispanic, White, or other/unknown. Investigators from each site recruited eligible patients according to the UK Brain Bank criteria for PD,^8^ the second consensus statement for MSA,^9^ and the Movement Disorders Society criteria for PSP.^10^ The site neurologist specializing in movement disorders performed a videotaped neurological and physical workup, obtained and evaluated standard clinical MRI scans (ie, T1- and T2-weighted MRI), administered clinical scales (Unified PD Rating Scale [UPDRS], Unified MSA Rating Scale [UMSARS], PSP Rating Scale [PSP-RS], etc), and reported a single diagnosis of PD, MSA (possible/probable), or PSP (possible/probable). Two independent offsite neurologists (T.X., M.O.) reviewed the videotaped examination, clinical MRIs, and clinical scales and provided their own blinded diagnosis. A unanimous agreement on the diagnosis from all 3 raters was required for study entry. A probable level of diagnostic certainty for MSA and PSP from at least 1 rater was needed.^9,10^ The AIDP analysis was performed after the clinical visit and was not used to guide diagnostic decisions. A retrospective auxiliary cohort was also included in this analysis, and the diagnosis for retrospective patients was determined by a neurologist specializing in movement disorders using established diagnostic criteria.^8,9,10^ However, diagnostic agreement among independent raters was not reported. Pathological diagnosis for a subset of patients who consented to brain donation was determined using established criteria by a single board-certified neuropathologist. Race and ethnicity data on the retrospective cohort were not available.

### MRI Acquisition and Processing

Diffusion MRI scans were obtained using Siemens, General Electric, and Philips 3-T scanners using a standard protocol^7^ (repetition time = 6000-13 000 milliseconds; echo time = 58-104 milliseconds; 90° flip angle; resolution = 2 mm isotropic; 80 interleaved slices; 0 mm gap; ≥30 directions; 5 b0 images; b = 1000 seconds/mm^2^). Two consecutive diffusion MRI scans were obtained to evaluate test-retest performance. Image processing was performed using FMRIB Software Library (University of Oxford) and Advanced Normalization Tools (open source). The pipeline included signal-to-noise calculation and quality control, motion and eddy-current correction, removal of nonbrain tissue, and standard space normalization. Custom MATLAB scripts were used to apply a 2-compartment model^11^ to calculate FW of the extracellular compartment and FW-corrected fractional anisotropy (FAt) of the tissue compartment^7,12,13^ (eMethods in Supplement 1). FW and FAt were calculated using custom atlases across 132 brain regions of interest that included the cortex, subcortex (basal ganglia, thalamic and limbic structures), brainstem, cerebellum, and transcallosal white matter.

### Machine Learning and Primary End Point Model

FW and FAt from brain regions of interest, as well as age and sex, composed an input feature vector for the linear kernel SVM.^7^ The training set included 78% of total patient data, which included 42% of the prospective cohort and 100% of the retrospective cohort. The remaining 58% of prospective data, representing 22% of total data, was reserved for independent testing. Prospective assignment to training and testing sets was achieved using stratified sampling to ensure that the proportion of each patient class was representative of the full cohort. All 21 sites were represented in both the training and testing sets. The retrospective cohort functioned to reinforce model training and was not used for independent testing. The training set was randomly split into 5 equally distributed validation subsets for 5-fold cross-validation. The model is trained using data from 4 subsets, and predictions are evaluated against the holdout subset. The process is repeated 5 times, and the model regularization parameter is adjusted at each fold to achieve optimal discrimination between the positive and negative diagnosis classes while balancing the trade-off between true- and false-positive rates. Feature reduction techniques were not used. The final model was then evaluated in the prospective independent testing set. There was no data leakage between the training and testing sets.

### Statistical Analysis

The discriminative performance of AIDP was determined using the area under the receiver operating characteristic curve (AUROC).^14^ The unanimous clinical diagnosis functioned as the reference ground truth unless postmortem neuropathology was available. Primary model end points included PD vs atypical parkinsonism, MSA vs PSP, PD vs MSA, and PD vs PSP. The AUROC 95% CIs were computed using the DeLong test.^15^ Model sensitivity, specificity, positive predictive value (PPV), and negative predictive value (NPV) were also calculated. The study was designed to have 80% power to detect a difference of 0.10 between the null (0.80) and alternative (0.90) AUROC using a 1-sided *z* test (α = .05). The Benjamini-Hochberg procedure for false discovery rate was applied to correct for multiple comparisons across end points. All *P* values were 2-sided, and *P* values <.05 were considered statistically significant.

We assessed the test-retest performance of the primary end points using the repeat diffusion scan. Additionally, the primary end points were tested with and without the inclusion of age and sex features.

We used linear regression to investigate whether diagnosis duration (ie, the time since parkinsonism diagnosis) and symptom severity (UPDRS III, UMSARS, PSP-RS) related to atypical probability estimates (ie, likelihood of atypical class assignment) for the primary end point model of PD vs atypical parkinsonism. We used diagnosis duration because a reported parkinsonism diagnosis was established before study entry, and patient memory of symptom onset time is subject to recall bias.

To further probe the performance of our primary end point model, we evaluated AIDP for discriminative performance variation when exposed to different training and testing sets across 49 additional runs. For each verification run, unique sets of prospective patients were assigned to the training and independent testing sets without data leakage. The same train/test split ratio and stratified sampling approach as the primary end point model was enforced for the verification runs. We reported the pooled AUROC performance (mean, 95% CI) for verification runs.

Next, we evaluated AIDP discriminative performance in prospective patients from 6 holdout sites not used in the model training. Stratified sampling was used to assign patients from 15 randomly selected sites to the training set and the remaining patients from 6 sites to the testing set. The proportion of PD, MSA, and PSP in the training and testing sets was representative of the full cohort. All retrospective patients were used in the training. We assessed AIDP variation over 49 verification runs using unique training and testing site assignments.

Finally, we evaluated AIDP discriminative performance of neuropathology cases for PD vs atypical parkinsonism and MSA vs PSP. The training set included prospective and retrospective patients without neuropathological confirmation. AIDP was evaluated on the independent test set of 49 neuropathology cases. Data were analyzed using Python, version 3.9.13 (Python Software Foundation).

## Results

### Participants

**Figure 1.**
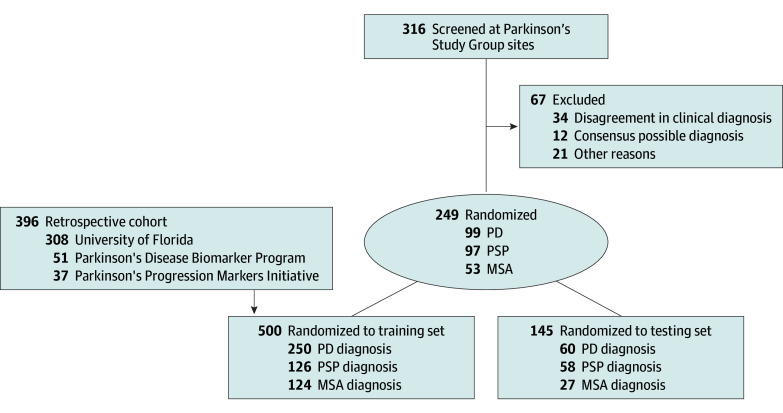
Enrollment and Allocation of Patients for Machine Learning Enrollment and allocation of patients to the training and testing sets for the primary end point model. Patients from the prospective cohort were assigned to training and testing sets using stratified sampling. Patients from the retrospective cohort were used to reinforce the training set and were not used in the independent testing of the model. MSA indicates multiple system atrophy parkinsonian variant; PD, Parkinson disease; PSP, progressive supranuclear palsy.

**Figure 2.**
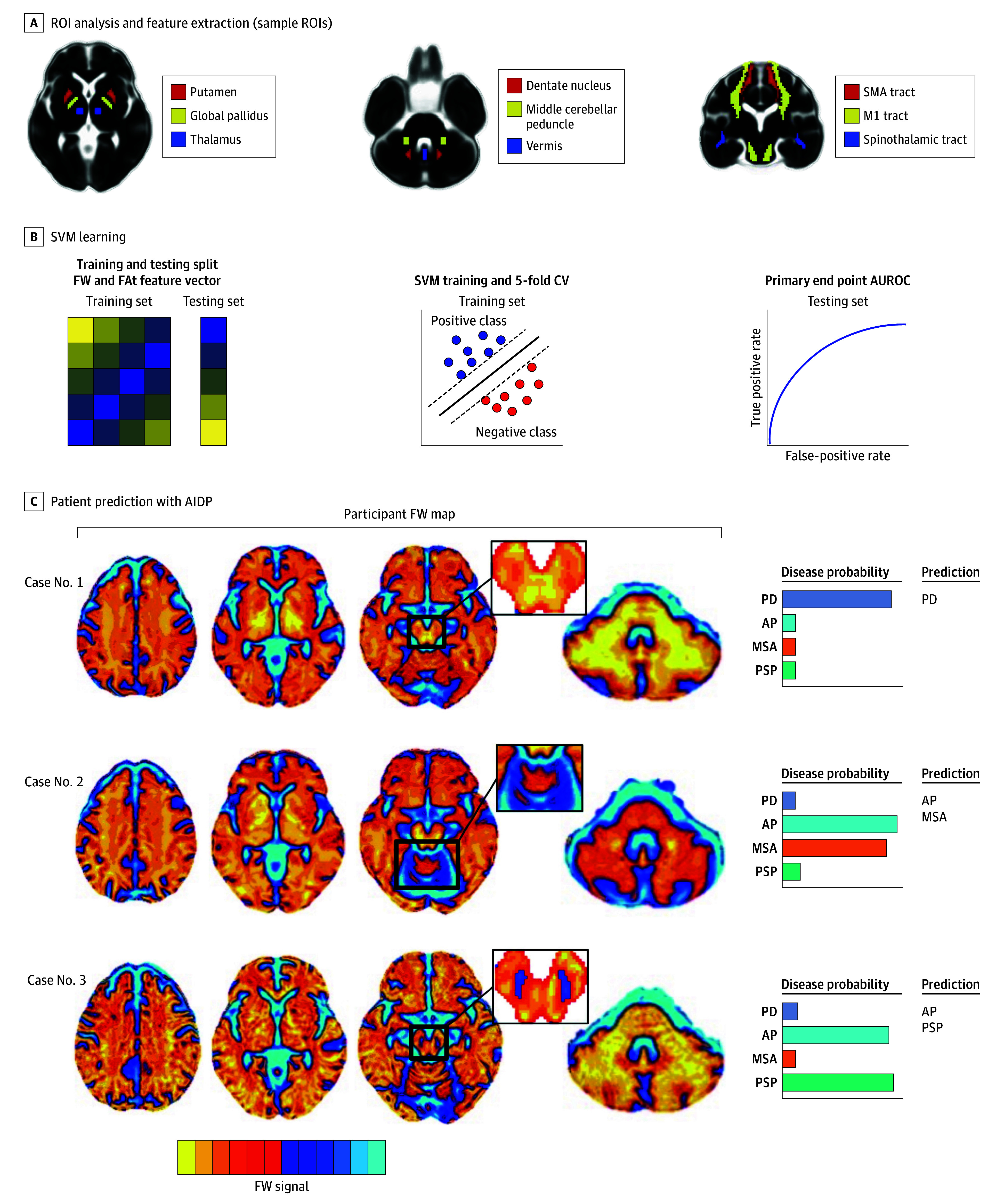
Automated Imaging Differentiation for Parkinsonism (AIDP) for Disease-Specific Classification of Parkinsonism A, Region of interest (ROI) analysis and feature extraction. Free-water (FW) and FW-corrected fractional anisotropy (FAt) values were calculated from 132 total brain ROIs. B, Support vector machine (SVM) learning. FW and FAt from brain ROIs, age, and sex composed a feature vector for the SVM input. The feature vector was split into a training set and independent testing set. Five-fold cross-validation (CV) was used during training to achieve the best possible discrimination between the positive and negative classes. The area under the receiver operating characteristic curve (AUROC) was obtained in the testing set for each primary end point (Parkinson disease [PD] vs atypical parkinsonism [AP], multiple system atrophy [MSA] parkinsonian variant vs progressive supranuclear palsy [PSP], PD vs MSA, and PD vs PSP). C, Patient predictions with AIDP. Three exemplar participant-level FW maps are shown for PD, MSA, and PSP testing cases. Higher FW levels are shown in blue/white colors. The corresponding disease-specific probability estimates and final AIDP diagnostic predictions are shown. M1 indicates primary motor cortex; SMA, supplementary motor area.

A total of 316 patients were eligible, and 67 patients were excluded due to diagnostic disagreement, unanimous determination of possible MSA or PSP, or MRI contraindications (Figure 1). The final prospective cohort included 249 patients (mean [SD] age, 67.8 [7.7] years; 94 female [37.8%]; 155 male [62.2%]; 14 Asian [5.6%]; 5 Black [2%]; 5 Hispanic [2%]; 244 non-Hispanic [98%]; 225 White [90.4%]; 5 other/unknown [2%]; 99 with PD, 53 with MSA, and 97 with PSP) from 21 sites (Table 1 and eTable 1 in Supplement 1). A retrospective auxiliary cohort of 396 patients (mean [SD] age, 65.8 [8.9] years; 162 female [40.9%]; 234 male [59.1%]; 211 with PD, 98 with MSA, and 87 with PSP) was also included in the analysis (Table 1 and eMethods in Supplement 1). The training set included 78% (n = 500) of the total patient data (250 with PD, 124 with MSA, and with 126 PSP), which included 104 prospective patients and all 396 retrospective patients. The remaining 145 patients (mean [SD] age, 67.4 [7.7] years; 50 female [34.5%]; 95 male [65.5%]) from the prospective cohort (60 with PD, 27 with MSA, and 58 with PSP), representing 22% of total data, were reserved for independent testing. The discriminative performance of AIDP was determined using the AUROC^14^ (Figure 2).

**Table 1.** Demographic and Clinical Characteristics^a^

Characteristic	No. (%)			
	Prospective cohort			Retrospective cohort, training set (N = 396)
	Total (N = 249)	Training set (n = 104)	Testing set (n = 145)	
Clinical diagnosis				
PD	99 (39.8)	39 (37.5)	60 (41.4)	211 (53.3)
MSA	53 (21.3)	26 (25.0)	27 (18.6)	98 (24.7)
PSP	97 (39.0)	39 (37.5)	58 (40.0)	87 (22.0)
Age, mean (SD), y				
All	67.8 (7.7)	68.4 (7.7)	67.4 (7.7)	65.8 (8.9)
PD	66.6 (7.9)	67.0 (8.5)	66.4 (7.6)	63.3 (8.9)
MSA	66.1 (9.4)	68.1 (9.2)	64.1 (9.3)	66.9 (8.7)
PSP	69.9 (6.0)	70.0 (5.5)	69.9 (6.4)	70.5 (6.9)
Sex				
Male	155 (62.2)	60 (57.7)	95 (65.5)	234 (59.1)
Female	94 (37.8)	44 (42.3)	50 (34.5)	162 (40.9)
Time since parkinsonism diagnosis, mean (SD), y				
All	3.7 (2.8)	3.7 (2.9)	3.6 (2.7)	NA
PD	6.3 (1.4)	6.2 (1.3)	6.4 (1.6)	NA
MSA	2.0 (2.3)	2.3 (3.0)	1.8 (1.3)	NA
PSP	1.9 (1.8)	2.2 (2.1)	1.7 (1.6)	NA
UPDRS part III, mean (SD)				
All	32.8 (15.9)	32.5 (16.4)	33.0 (15.6)	37.6 (19.6)
PD	22.0 (10.3)	20.8 (11.9)	22.8 (9.1)	27.8 (13.1)
MSA	40.1 (15.3)	38.5 (14.8)	41.6 (16.0)	55.7 (18.7)
PSP	39.8 (14.9)	40.2 (14.7)	39.5 (15.1)	41.0 (18.1)
UMSARS total, mean (SD)				
All	35.0 (18.2)	34.0 (18.3)	35.7 (18.2)	NA
PD	19.1 (7.4)	17.3 (7.8)	20.4 (6.9)	NA
MSA	46.9 (16.8)	42.8 (15.9)	50.9 (16.9)	NA
PSP	44.7 (14.8)	45.0 (14.7)	44.5 (14.9)	NA
PSP-RS total, mean (SD)				
All	26.0 (17.1)	25.5 (17.2)	26.3 (17.1)	NA
PD	10.5 (5.0)	9.7 (5.2)	10.9 (4.8)	NA
MSA	28.5 (12.4)	26.9 (12.4)	30.1 (12.4)	NA
PSP	40.4 (13.7)	40.3 (14.0)	40.5 (13.5)	NA

Abbreviations: MSA, multiple system atrophy parkinsonian variant; NA, not available; PD, Parkinson disease; PSP, progressive supranuclear palsy; PSP-RS, PSP Rating Scale; UMSARS, Unified MSA Rating Scale; UPDRS, Unified PD Rating Scale.

^a^ Demographic and clinical information for the prospective and retrospective cohorts used in the AIDP primary end point model. The prospective cohort is further stratified by training and testing sets. The retrospective cohort was only used in the training set. Additional clinical scales for the prospective cohort are provided in eTable 2 in Supplement 1.

The pathological subset collected over 3 years since study initiation included 5 PD, 5 MSA, and 39 PSP brains. Brains from 4 patients (2 MSA, 1 PSP, 1 PD) were drawn from the prospective cohort (Table 1 and eTable 2 in Supplement 1).

### AIDP Classification of Primary End Points

**Figure 3.**
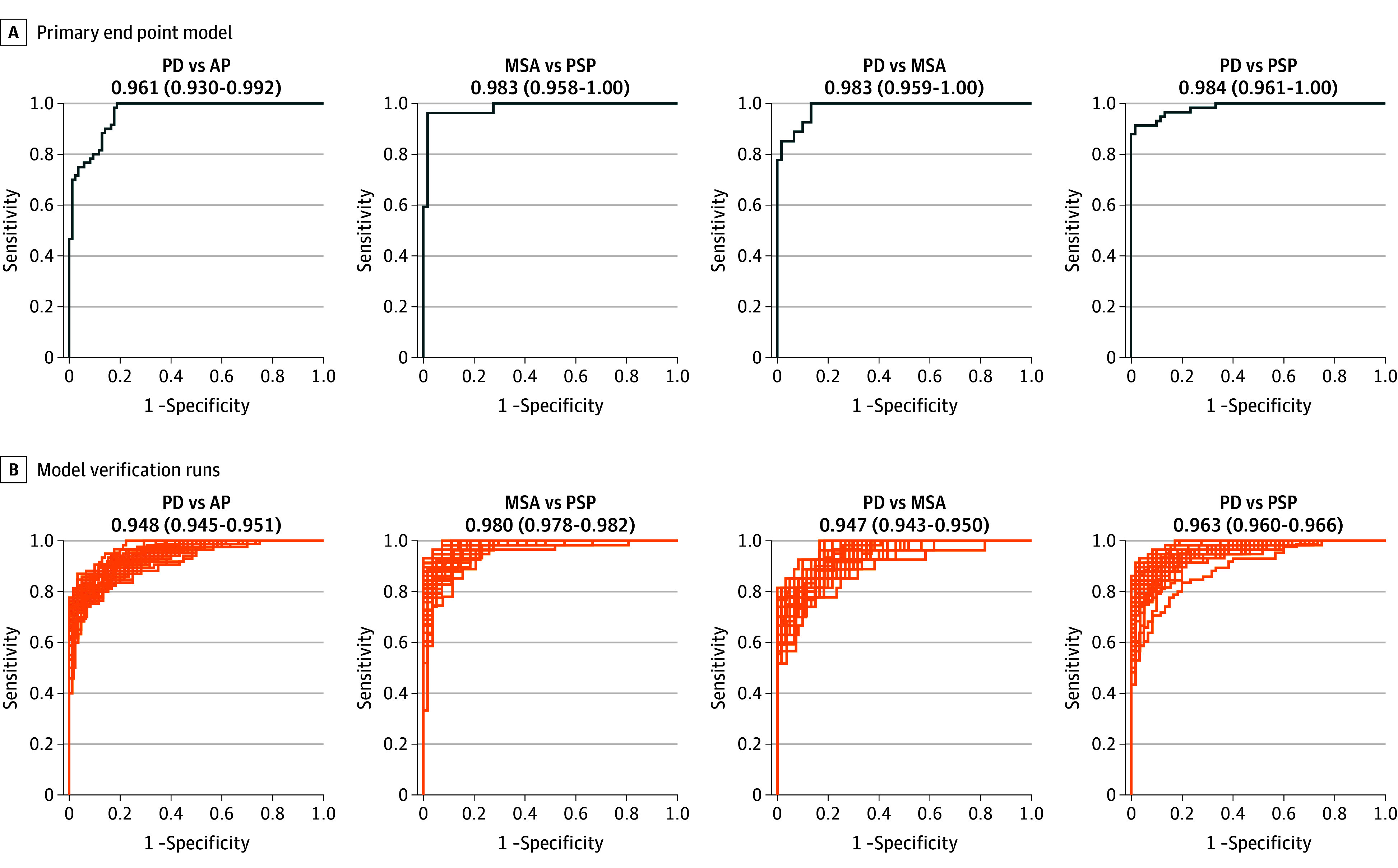
Automated Imaging Differentiation for Parkinsonism (AIDP) Primary End Point Model and Verification Run Performance A, Area under the receiver operating characteristic curve (AUROC) for the primary model end points of Parkinson disease (PD) vs atypical parkinsonism (AP), multiple system atrophy parkinsonian variant (MSA) vs progressive supranuclear palsy (PSP), PD vs MSA, and PD vs PSP. The AUROC and 95% CI calculated using the DeLong method are reported for each end point. B, AUROC for 49 model verification runs for each primary end point. The average AUROC across all runs and the 95% CI on the mean are reported.

The primary end point model achieved high AUROCs for PD vs atypical parkinsonism, 0.96 (95% CI, 0.93-0.99; sensitivity, 0.87; specificity, 0.88; PPV, 0.91; NPV, 0.83; *P* <.001), MSA vs PSP, 0.98 (95% CI, 0.96-1.00; sensitivity, 0.90; specificity, 0.96; PPV, 0.98; NPV, 0.81; *P* <.001), PD vs MSA, 0.98 (95% CI, 0.96-1.00; sensitivity, 0.97; specificity, 0.85; PPV, 0.97; NPV, 0.97; *P* <.001), and PD vs PSP, 0.98 (95% CI, 0.96-1.00; sensitivity, 0.98; specificity, 0.91; PPV, 0.92; NPV, 0.98; *P* <.001) ( Table 2, Figure 3A). Retest AUROCs using the repeat diffusion scan revealed comparable performance (PD vs atypical parkinsonism, 0.94; 95% CI, 0.90-0.98; *P* <.001; MSA vs PSP, 0.98; 95% CI, 0.95-1.00; *P* <.001; PD vs MSA, 0.94; 95% CI, 0.89-0.99; *P* <.001; and PD vs PSP, 0.98; 95% CI, 0.96-1.00; *P* <.001) (eFigure 1 in Supplement 1). Additionally, the elimination of age and sex features did not adversely affect model performance (PD vs atypical parkinsonism, 0.96; 95% CI, 0.93-0.99; *P* <.001; MSA vs PSP, 0.98; 95% CI, 0.96-1.00; *P* <.001; PD vs MSA, 0.98; 95% CI, 0.95-1.00; *P* <.001; and PD vs PSP, 0.99; 95% CI, 0.96-1.00; *P* <.001) (eFigure 2 in Supplement 1). The pooled performance of 49 additional verification runs revealed high average testing AUROCs for PD vs atypical parkinsonism, 0.95 (95% CI, 0.95-0.95; *P* <.001), MSA vs PSP, 0.98 (95% CI, 0.98-0.98; *P* <.001), PD vs MSA, 0.95 (95% CI, 0.94-0.95; *P* <.001), and PD vs PSP, 0.96 (95% CI, 0.96-0.97; *P* <.001) (Table 2, Figure 3B). Thus, AIDP was reliable for predicting primary end points when exposed to unique training and testing sets.

**Table 2.** Automated Imaging Differentiation for Parkinsonism (AIDP) Training, Validation, and Testing Metrics^a^

Metric	Primary end point model			Pooled verification run performance		
	Training	Validation	Testing	Training	Validation	Testing
**PD vs AP**						
AUROC	0.916	0.917	0.961	0.920 (0.914-0.926)	0.860 (0.851-0.869)	0.948 (0.945-0.951)
Sensitivity	0.876	0.857	0.871	0.884 (0.875-0.894)	0.814 (0.798-0.830)	0.858 (0.851-0.866)
Specificity	0.956	0.977	0.883	0.956 (0.951-0.960)	0.906 (0.895-0.917)	0.867 (0.854-0.879)
Positive predictive value	0.950	0.980	0.914	0.952 (0.947-0.957)	0.897 (0.884-0.909)	0.902 (0.894-0.910)
Negative predictive value	0.891	0.843	0.828	0.893 (0.884-0.901)	0.830 (0.816-0.843)	0.813 (0.805-0.820)
**MSA vs PSP**						
AUROC	0.971	0.857	0.983	0.982 (0.977-0.987)	0.883 (0.872-0.895)	0.980 (0.978-0.982)
Sensitivity	0.951	0.826	0.897	0.979 (0.973-0.986)	0.866 (0.845-0.888)	0.879 (0.868-0.889)
Specificity	0.990	0.888	0.962	0.984 (0.979-0.989)	0.900 (0.881-0.920)	0.942 (0.933-0.950)
Positive predictive value	0.990	0.864	0.981	0.984 (0.979-0.989)	0.906 (0.889-0.923)	0.970 (0.966-0.975)
Negative predictive value	0.950	0.857	0.813	0.980 (0.974-0.986)	0.863 (0.842-0.884)	0.787 (0.772-0.802)
**PD vs MSA**						
AUROC	0.984	0.945	0.983	0.976 (0.973-0.980)	0.975 (0.969-0.981)	0.947 (0.943-0.950)
Sensitivity	0.976	0.933	0.967	0.943 (0.937-0.950)	0.950 (0.939-0.961)	0.858 (0.845-0.871)
Specificity	0.977	0.889	0.852	0.936 (0.926-0.946)	0.937 (0.923-0.951)	0.842 (0.830-0.854)
Positive predictive value	0.990	0.914	0.967	0.968 (0.963-0.973)	0.967 (0.959-0.975)	0.858 (0.845-0.871)
Negative predictive value	0.945	0.913	0.967	0.892 (0.880-0.903)	0.906 (0.886-0.926)	0.858 (0.845-0.871)
**PD vs PSP**						
AUROC	0.945	0.891	0.984	0.963 (0.956-0.970)	0.886 (0.873-0.899)	0.963 (0.960-0.966)
Sensitivity	0.960	0.902	0.983	0.978 (0.974-0.982)	0.931 (0.919-0.943)	0.948 (0.939-0.957)
Specificity	0.931	0.880	0.914	0.948 (0.938-0.958)	0.841 (0.820-0.862)	0.843 (0.831-0.855)
Positive predictive value	0.965	0.934	0.922	0.974 (0.969-0.979)	0.916 (0.904-0.928)	0.863 (0.855-0.872)
Negative predictive value	0.922	0.815	0.981	0.956 (0.948-0.964)	0.866 (0.847-0.885)	0.941 (0.932-0.951)

Abbreviations: AP, atypical parkinsonism; AUROC, area under the receiver operating characteristic curve; MSA, multiple system atrophy parkinsonian variant; PD, Parkinson disease; PSP, progressive supranuclear palsy.

^a^ The training metrics represent the model’s discriminative performance on the data it was trained on (ie, the full training set). The validation metrics are obtained from the best-performing cross-validation fold in the training set. The testing metrics represent the model’s discriminative performance on the independent testing set (ie, data not used in the training).

### Effects of Diagnosis Duration and Symptom Severity on AIDP Classification

We assessed whether the diagnosis duration and symptom severity for patients with MSA and PSP related to the likelihood of atypical class assignment for the primary end point model of PD vs atypical parkinsonism. Patients with MSA and PSP in the testing set had a mean (SD) diagnosis duration of 1.8 (1.3) and 1.7 (1.6) years, respectively. The majority had a diagnosis duration under 3 years (MSA, 85%; PSP, 79%). The regression between diagnosis duration and atypical probability estimates did not reach statistical significance for MSA (*R*^2^ = 0.05; *P* = .50) or PSP (*R*^2^ = 0.05; *P* = .43) (eFigure 3 in Supplement 1). Atypical probability estimates for MSA were positively associated with UPDRS III (*R*^2^ = 0.337; *P* = .003) and UMSARS (*R*^2^ = 0.43; *P* < .001) scores, suggesting that patients with MSA and worsened symptom severity were more likely to be classified as the true class. Atypical probability estimates for PSP cases were not significantly moderated by symptom severity scores for UPDRS III (*R*^2^ = 0.10; *P* = .06) or PSP-RS (*R*^2^ = 0.03; *P* = .21).

### AIDP Classification With Site Preservation

The AIDP site preservation model achieved high AUROCs in the independent testing set of 6 holdout sites for PD vs atypical parkinsonism, 0.94 (95% CI, 0.89-0.99; *P* <.001), MSA vs PSP, 0.94 (95% CI, 0.87-1.00; *P* <.001), PD vs MSA, 0.96 (95% CI, 0.91-1.00; *P* <.001), and PD vs PSP, 0.93 (95% CI, 0.92-1.00; *P* <.001) (eFigure 4 in Supplement 1). The pooled performance of 49 additional verification runs revealed high average testing AUROCs for PD vs atypical parkinsonism, 0.90 (95% CI, 0.89-0.91; *P* <.001), MSA vs PSP, 0.96 (95% CI, 0.95-0.97; *P* <.001), PD vs MSA, 0.87 (95% CI, 0.86-0.89; *P* <.001), and PD vs PSP, 0.94 (95% CI, 0.93-0.95; *P* <.001). The findings indicate that AIDP generalized to new site data.

### AIDP Classification of Neuropathology

The median time between the last clinical imaging scan and autopsy in the pathological subset was 34 months (range, 4.8-94.8 months). The neuropathology model achieved high testing AUROCs for PD vs atypical parkinsonism, 0.99 (95% CI, 0.96-1.00; *P* = .001) and MSA vs PSP, 0.97 (95% CI, 0.93-1.00; *P* = .001) (eFigure 5 in Supplement 1). The AIDP diagnosis was confirmed neuropathologically in 46 of 49 brains (93.9%; 5 of 5 PD, 5 of 5 MSA, and 36 of 39 PSP), representing a 12.3% diagnostic gain compared with the last clinical diagnosis (81.6%; 4 of 5 PD, 3 of 5 MSA, 33 of 39 PSP). AIDP predicted the neuropathology in all 4 brains from the prospective cohort (2 MSA, 1 PSP, 1 PD). This included 1 case with unanimous possible PSP from the clinical diagnosis and later confirmed pathological MSA and 1 case with unanimous possible MSA from the clinical diagnosis and later confirmed pathological Lewy body disease (eg, PD).

## Discussion

This prospective multicenter cohort study of AIDP met its primary end points. The primary end point model AUROC was 0.96 for PD vs atypical parkinsonism and greater than 0.98 for MSA vs PSP, PD vs MSA, and PD vs PSP. Overall confidence in the results was bolstered by solid performance in the test-retest analysis, the revised model without age and sex, and the model verification runs. Additionally, AIDP was successfully validated against site holdout and autopsy cases. Finally, the successful application across 11 Siemens, 5 General Electric, and 5 Philips 3-T scanners supports the potential for widespread implementation of AIDP software, which is designed for cloud-based integration with picture archiving and communication systems (eFigure 6 in Supplement 1).

DaT SPECT, skin biopsy, and synuclein seed aggregation assay (SAA) have all been proposed to aid in diagnosing PD.^16^ DaT SPECT as a biomarker for PD has been shown to demonstrate high sensitivity (98%) but low specificity (67%).^6^ Similarly, the cerebrospinal fluid–based SAA has demonstrated high sensitivity for PD.^17,18^ Sensitivity was 98.6% for PD with typical olfactory deficit and dropped to 78.3% in those without olfactory loss and approximately 68% when *LRRK2* gene variants were present.^17^ However, *LRRK2*-PD can occur in the absence of Lewy body pathology.^19^ A serum-based biomarker called immunoprecipitation real-time quaking-induced conversion has been recently introduced, demonstrating greater than 0.90 AUROC for PD vs controls and 0.64 to 0.73 AUROC for MSA vs controls.^20,21^ Additionally, Gibbons and colleagues^22^ detected phosphorylated α-synuclein using skin biopsy in the majority of patients with PD, MSA, dementia with Lewy bodies, and pure autonomic failure. However, these assays have not been shown to reliably differentiate between PD and MSA. Further, none of the aforementioned assays are specific for PSP. The AIDP primary end point model differentiated both MSA and PSP from PD with AUROC greater than 0.98, PPV greater than 92%, and NPV greater than 96%.

Diagnosis remains critical for appropriate clinical treatment, as well as for inclusion in clinical trials. The PD field has begun considering a biological classification and staging system, such as the recently adopted ATN (ie, amyloid, tau, neurodegeneration) system for Alzheimer disease.^23^ The proposed framework relies on evidence of pathological neuronal α-synuclein and dopaminergic neuron degeneration as core biological anchors, regardless of the clinical syndrome.^16^ FW imaging has proven capable of providing quantitative longitudinal information on disease-specific neurodegeneration between PD, MSA, and PSP.^24^ It is possible that the future application of AIDP, in combination with other neuronal α-synuclein biomarkers, may be a useful component of the PD classification and staging system.

Clinical-ready biomarkers are critically needed to steer clinicians toward a more specific diagnosis during a patient workup. In clinicopathological studies from the Queen Square Brain Bank, the PPV for MSA pathology was greater than 90% for clinically probable MSA and approximately 71% to 75% for clinically possible MSA.^25,26^ Additionally, Koga and colleagues found that 42 of 134 patients (31.3%) with clinically probable or possible MSA had Lewy body disease or PSP.^27^ Similarly, a clinical PPV of 78% was reported for PSP, with Lewy body disease and MSA as prominent pathologies among patients with a false-positive clinical diagnosis.^28^ In the current study, the AIDP diagnosis was confirmed neuropathologically in 93.9% of autopsy cases, whereas clinical diagnosis was confirmed in 81.6%. Further, an important finding was that AIDP predicted the neuropathology in 2 cases with a unanimous possible—but incorrect—clinical diagnosis from 3 expert raters.

### Strengths and Limitations

Strengths of the current study included a large and well-powered sample, the use of prospectively obtained testing data, and the requirement for diagnostic agreement among 3 experts.

There are also several limitations that deserve discussion. In the current study, a robust ground-truth diagnosis was established by 3 independent raters. In the future, we will analyze cases with clinical ambiguity and rater disagreement and compare AIDP with neuropathology as more cases become available. Although the majority of postmortem brains were from patients with PSP, we anticipate more MSA and PD brains will become available to expand the pathological validation of AIDP. Finally, future studies should consider prodromal cases, cases of dementia with Lewy bodies and corticobasal syndrome, and cases from clinical settings outside of specialist movement disorders centers.

## Conclusions

In conclusion, in this prospective multicenter cohort study, the successful testing and validation of AIDP suggest its integration within the diagnostic workup of 3 commonly encountered neurodegenerative parkinsonian disorders. The combination of AIDP plus SAA, skin biopsy, or both may offer a more practical, affordable, and accessible approach for diagnosis and disease staging.

## Notes

Supplement 1.
eMethods.
**eTable 1.** Characteristics of the Prospective Cohort by Parkinson Study Group Site **eTable 2.** Clinical Scales for the Prospective Cohort **eFigure 1.** AIDP Retest of Primary End Point Model **eFigure 2.** AIDP Primary and Secondary End Points for the Diffusion Only Model **eFigure 3.** Regression Analyses for Diagnosis Duration and Symptom Severity Scores With Atypical Probability Estimates **eFigure 4.** AIDP Site Preservation Model for Primary End Points **eFigure 5.** AIDP Neuropathology Model End Points **eFigure 6.** Use of AIDP in a Diagnostic Patient Workup
eReferences

Supplement 2.
Data Sharing Statement.
