# Supplementary material for: Automated Imaging Differentiation for Parkinsonism
Source: JAMA Neurol. 2025 Mar 17;82(5):495–505. doi: 10.1001/jamaneurol.2025.0112 (PMC11915115; doi:10.1001/jamaneurol.2025.0112)
Supplement: Supplement 1. — eMethods. eTable 1. Characteristics of the Prospective Cohort by Parkinson Study Group Site eTable 2. Clinical Scales for the Prospective Cohort eFigure 1. AIDP Retest of Primary End Point Model eFigure 2. AIDP Primary and Secondary End Points for the Diffusion Only Model eFigure 3. Regression Analyses for Diagnosis Duration and Symptom Severity Scores With Atypical Probability Estimates eFigure 4. AIDP Site Preservation Model for Primary End Points eFigure 5. AIDP Neuropathology Model End Points eFigure 6. Use of AIDP in a Diagnostic Patient Workup eReferences [file jamaneurol-e250112-s001.pdf]

Supplementary Online Content

Vaillancourt DE, Barmpoutis A, Wu SS, et al; AIDP Study Group. Automated imaging differentiation for parkinsonism. *JAMA Neurol*. Published online March 17, 2025. doi:10.1001/jamaneurol.2025.0112

eMethods.

eTable 1. Characteristics of the Prospective Cohort by Parkinson Study Group Site

eTable 2. Clinical Scales for the Prospective Cohort

eFigure 1. AIDP Retest of Primary End Point Model

eFigure 2. AIDP Primary and Secondary End Points for the Diffusion Only Model

eFigure 3. Regression Analyses for Diagnosis Duration and Symptom Severity Scores With Atypical Probability Estimates

eFigure 4. AIDP Site Preservation Model for Primary End Points

eFigure 5. AIDP Neuropathology Model End Points

eFigure 6. Use of AIDP in a Diagnostic Patient Workup

eReferences

This supplementary material has been provided by the authors to give readers additional information about their work.

© 2025 Vaillancourt DE et al. *JAMA Neurology*.

eMethods

Device Description and Indications for Use Statement

Automated Imaging Differentiation for Parkinsonism (AIDP) is a machine learning software that is intended to assist neurologists, radiologists, and other physicians in differentiating between Parkinson's disease (PD), multiple system atrophy parkinsonian variant (MSA), and progressive supranuclear palsy (PSP). The user-provided input is a single-shell diffusion-weighted MRI scan acquired using widely available 3 Tesla scanners from major vendors (Siemens, General Electric, Philips). AIDP can be integrated as a plugin with picture archiving and communication systems and medical image viewers. An application programming interface (API) allows users to authenticate, upload data, and retrieve results across platforms. The software receives the image in DICOM format using a secure data uploading and encrypted transmission process. The software analyzes the image using a machine learning algorithm and provides a report to assist in clinical diagnostic decision-making. AIDP is intended for patients aged 40 years and older presenting with clinical symptoms of parkinsonism. The results are intended to provide supplemental information in conjunction with a standard neurological assessment and other clinical tests. Patient management decisions should not be made solely on the basis of the AIDP analysis.

Participants

Prospective Cohort of the Parkinson's Study Group

Patients were recruited and enrolled at 21 specialist movement disorders centers of the Parkinson's Study Group. The AIDP Steering Committee consisting of six expert movement disorders neurologists chose the 21 sites out of 88 possible. Sites were ranked and selected based on geographic location, reputation for recruitment and neurological care, and MRI facilities and equipment. The final sites included 19 centers located in the United States and two in Canada. From July 2021 to January 2024, 316 patients were screened, 67 were excluded, and 249 were enrolled in the study (99 PD, 53 MSA, 97 PSP). The characteristics of the prospective cohort by Parkinson's Study Group site are provided in eTable 1.

1. Albany Medical College
2. Augusta University
3. Beth Israel Deaconess Medical Center
4. Duke University Medical Center
5. John Hopkins University
6. Massachusetts General Hospital
7. Northwestern University
8. Ottawa Hospital Research Institute
9. Pennsylvania State University
10. University of Alabama of Birmingham
11. University of California San Diego
12. University of California San Francisco
13. University of Chicago
14. University of Florida
15. University of Kentucky
16. University of Michigan
17. University of Minnesota
18. University of South Florida
19. University of Toronto, Centre for Addiction and Mental Health
20. Wake Forest University
21. Washington University in St. Louis

Inclusion Criteria

- 40 – 80 years of age
- PD diagnosis according to the UK PD brain bank criteria[1,2] with a symptom duration of at least five years, Hoehn and Yahr stage 2 or 3 on medication
- Probable PSP diagnosis according to the Movement Disorders Society criteria[3]
- Probable MSA diagnosis according to the Second Consensus Statement for MSA[4]
- Ability to undergo MRI
- Capable of reading and understanding English and has provided written informed consent to participate

Exclusion Criteria

- Diagnostic disagreement among blinded raters
- Unanimous determination of a possible diagnosis for MSA or PSP
- Stroke, brain tumor, seizures, or other serious neurological condition
- MRI contraindications (e.g., pacemaker, neurostimulator, aneurysm clip, etc.)

© 2025 Vaillancourt DE et al. *JAMA Neurology*.

- Women who are or might be pregnant or nursing

Retrospective Cohort
Retrospective data from three databases were included to help reinforce the training of the AIDP machine learning model. Retrospective data were not used in the independent testing of the AIDP model.

1. University of Florida Legacy Dataset (contributed to PDBP and PPMI), PI: David Vaillancourt, Ph.D.
2. Parkinson's Disease Biomarker Program (PDBP), PIs: Christine Swanson-Fischer, Ph.D., Debra Babcock, M.D., Ph.D.
3. Parkinson's Progression Marker Initiative (PPMI), PI: Ken Marek, M.D.

Pathological Subset
The **pathological subset** collected over three years since study initiation included 5 PD, 5 MSA, and 39 PSP brains. The majority of brains were obtained from National Alzheimer's Coordinating Center (NACC), Parkinson's Progression Marker Initiative (PPMI), and 4 Repeat Tauopathy Neuroimaging Initiative (4RTNI). Brains from four patients (two MSA, one PSP, one PD) were drawn from the prospective 21-site cohort of the Automated Imaging Differentiation for Parkinsonism study.

1. National Alzheimer's Coordinating Center (NACC), PI: Walter Kukhill, Ph.D.
2. Parkinson's Progression Marker Initiative (PPMI), PI: Ken Marek, M.D.
3. 4 Repeat Tauopathy Neuroimaging Initiative (4RTNI), PI: Adam Boxer, MD
4. Automated Imaging Differentiation for Parkinsonism (Prospective Cohort), PI: David Vaillancourt, Ph.D.

Clinical Procedure for Patient Diagnosis (Reference Ground Truth for Machine Learning)
Investigators from each site recruited eligible patients according to the UK Brain Bank criteria for PD[2], the second consensus statement for MSA[4], and the Movement Disorders Society criteria for PSP[3]. The site movement disorders neurologist performed a videotaped neurological and physical workup, obtained and evaluated standard clinical MRI scans (i.e., T1- and T2-weighted MRI), administered clinical scales (provided below), and reported a single diagnosis of PD, possible MSA, probable MSA, possible PSP, or probable PSP. The video protocol allowed the independent neurologists to visualize the neurological evaluation. The neurological video protocol was developed by the Steering Committee, including Dr. Nick McFarland, Dr. Michael Okun, Dr. Irene Litvan, Dr. Hubert Fernandez, Dr. Tao Xie, Dr. Alex Pentalyat, and Dr. Robert Hauser. Two additional offsite movement disorders neurologists (Dr. Tao Xie and Dr. Michael Okun) reviewed the clinical information, neurological video, and standard brain MRI. The independent offsite neurologists each provided their diagnosis while blinded to the diagnosis of the site neurologist and each other. Patients were enrolled in the study if there was unanimous agreement in the patient diagnosis from all three neurologists. That is, all neurologists independently and blindly arrived at the same diagnosis. A probable level of diagnostic certainty from at least one rater was required for MSA and PSP[3,4]. There was no consensus meeting among neurologists to discuss individual interpretations of the clinical information and refine the diagnosis. Patients were not enrolled if there was diagnostic dissent among blinded raters or if there was a unanimous determination of possible MSA or PSP. The unanimous clinical diagnosis was used as the reference gold standard for training and testing the AIDP machine learning model. All patients were approached about consenting for brain donation to the University of Florida Neuromedicine Brain Bank led by Dr. Stefan Prokop, a board-certified neuropathologist. The pathological diagnosis served as the reference ground truth in deceased patients who consented to brain donation.

Clinical Evaluations
Below are the clinical scales and assessments performed at each site. The referring site neurologists administered the clinical scales, and all patients were assessed in an off-medication state.

- Demographic information and medical history
- Neurological screening and vitals form (cranial nerves and reflexes + BPs in seated, standing, and supine positions)
- Unified Parkinson's Disease Rating Scale (UDPRS)
- Progressive Supranuclear Palsy Rating Scale (PSP-RS)
- Unified Multiple System Atrophy Rating Scale (UMSARS)
- Epworth Sleepiness Scale
- Hamilton Anxiety
- Hamilton Depression
- Montreal Cognitive Assessment (MoCA)
- Modified Schwab and England Activities of Daily Living
- Parkinson's Disease Quality of Life Questionnaire (PDQ39)
- Rapid Eye Movement Behavior Disorder Questionnaire

© 2025 Vaillancourt DE et al. *JAMA Neurology*.

MRI Acquisition
MRI scans were acquired using Siemens, Philips, and General Electric 3 Tesla clinical MRI scanners. Whole brain diffusion MRI was obtained with a single-shot echo planar imaging sequence with the following acquisition parameters:

- TR between 6000 ms – 13000 ms
- TE between 58 ms – 104 ms
- Flip angle 90 degrees
- 2 mm isotropic voxels
- $\geq$ 80 Interleaved slices with zero gap
- $\geq$ 30 directions
- B0 images = 5
- Max b-value 1000 s/mm^2

In addition, a phantom fBIRN was acquired to assess the non-patient image and scanner quality for each site and on each day a participant was scanned. Standard clinical T1- and T2-weighted MRI scans were obtained to normalize the diffusion MRI.

The acquired MRI scans were provided in DICOM imaging format (Digital Imaging and Communications in Medicine). The header of the DICOM images contained the following tags:

- [0008, 0070] Scanner name
- [2005, 1415][a] [2005, 1599][a] b vectors
- [0018, 1312][b] phase encoder
- [0019, 10bb][b] [0019, 10bc][b] [0019, 10bd][b] b vectors
- [0019, 10e0][b] b directions
- [0019, 100c][c] b value
- [0019, 100d][c] diffusion directionality
- [0019, 100e][c] b directions
- [0019, 1027][c] b matrix
- [0043, 1039][b] b value

[a]: usually used by Philips scanners
[b]: usually used by GE scanners
[c]: usually used by SIEMENS scanners.

The input images were de-identified by removing the tags with sensitive information from the DICOM header, including but not limited to the following tags:

- [0008, 0080] Institution Name
- [0008, 0081] Institution Address
- [0008, 0082] Institution Code Sequence
- [0008, 0090] Referring Physician Name
- [0008, 0092] Referring Physician Address
- [0008, 0094] Referring Physician Telephone Numbers
- [0008, 0096] Referring Physician Identification Sequence
- [0008, 1040] Institutional Department Name
- [0008, 1048] Physicians of Record
- [0008, 1049] Physicians of Record Identification Sequence
- [0008, 1050] Performing Physician Name
- [0008, 1052] Performing Physician Identification Sequence
- [0008, 1060] Name of Physicians Reading Study
- [0008, 1062] Physicians Reading Study Identification Sequence
- [0008, 1070] Operators Name
- [0008, 1072] Operator Identification Sequence
- [0008, 1080] Admitting Diagnoses Description
- [0008, 1084] Admitting Diagnoses Code Sequence
- [0008, 1050] Performing Physician Name
- [0010, xxxx] Patient’s information
- [0032, xxxx] Medical visit details
- [0040, 0006] Scheduled performing physician’s name

© 2025 Vaillancourt DE et al. *JAMA Neurology*.

Free-water Image Processing

Diffusion image processing is controlled using custom Linux/Unix shell scripts operating FMRIB Software Library (FSL) (https://fsl.fmrib.ox.ac.uk/fsl/fslwiki) and Advanced Normalization Tools (ANTs) (http://stnava.github.io/ANTs/)[5–7]. All scans are visually inspected and confirmed to be devoid of acquisition artifacts and lesions such as stroke or tumor prior to processing. The diffusion image processing pipeline[8] includes signal-to-noise calculation of the b=0 and b=1000 images, motion and eddy-current correction, removal of non-brain tissue, and normalization to standard space. Custom MATLAB scripts are used to apply a two-compartment free-water model to each voxel[8]. The extracellular compartment is described by a single parameter, the **fractional volume of free water (FW)**, measured on a scale of 0 to 1 (more isotropic)[9]. The **fractional anisotropy of the tissue compartment (FAt)** is obtained from the diffusion tensor corrected for FW-related signal attenuation. FAt is measured on a scale of 0 to 1 (more anisotropic). FW and FAt were calculated across 132 brain regions of interest, including the cortex, subcortex (basal ganglia, thalamic and limbic structures), brainstem, cerebellum, and transcallosal white matter using custom atlases.

Support Vector Machine Learning

AIDP uses a support vector machine (SVM) with a linear kernel implemented in Python using the scikit-learn library[11]. A linear kernel indicates that the data points are represented using their original feature space instead of being projected into a high-dimensional space for classification. FW and FAt values from 132 brain regions of interest, as well as age and sex, compose an input feature vector for the machine learning model. The training set included 78% (n=500) of total patient data (250 PD,124 MSA, 126 PSP), which included 104 prospective patients and all 396 retrospective patients. Thus, 42% of the prospective data were used to train the model. The remaining 60% of prospective data (n=145), representing 22% of the total combined data, were reserved for independent testing of the model. The retrospective cohort functioned to reinforce model training and was not used for independent testing. Thus, the model was only tested on prospectively obtained data with unanimous agreement in the diagnosis from three expert raters. There was no data leakage, as the training and testing sets were kept separate.

Five-fold cross-validation was used during the model training to optimize the decision hyperplane (or decision boundary) that separates diagnosis classes. The model is trained using data from four subsets (k-1 folds), and predictions are evaluated against the remaining holdout subset. The process is repeated five times, and the model regularization parameter is adjusted at each fold to achieve optimal discrimination between the positive and negative diagnosis classes while balancing the trade-off between true and false positive rates. The F1 score (i.e., the harmonic mean of the precision and recall) is calculated at each fold and helps fine-tune the regularization parameter. The final optimized model was then evaluated in the prospective independent testing set. Feature reduction techniques (e.g., recursive feature elimination, Lasso) were not used to fine-tune the model.

A receiver-operating characteristic (ROC) analysis was used to evaluate the discriminative performance of the model in the independent testing dataset for primary model endpoints (PD versus atypical parkinsonism, MSA versus PSP, PD versus MSA, and PD versus PSP). The AUC is determined by evaluating the tradeoff between sensitivity and specificity at various thresholds (or probability cutoffs) used for positive class assignment. The threshold for positive class assignment (probability >0.5) was defined *a priori*. For example, in the primary model endpoint of PD versus atypical parkinsonism, a probability >0.5 was weighted toward atypical parkinsonism (positive class), whereas a probability < 0.5 was weighted toward PD (negative class). The main summary statistic for the model was the area under the curve (AUC) for each primary endpoint. Sensitivity, specificity, positive predictive value, and negative predictive value were calculated from the confusion matrix.

The study was designed to have 80% power to detect a difference of 0.10 between the null (0.80) and alternative (0.90) AUC using a one-sided z-test (α=0.05). The null AUC is set at 0.800 because that is the tolerable AUC for clinical applications and indicates high classification capacity and strong effect size.

© 2025 Vaillancourt DE et al. *JAMA Neurology*.

eTable 1. Characteristics of the prospective cohort by Parkinson's Study Group site.

Characteristic	Albany (US)	Augusta (US)	Beth Israel (US)	Duke (US)	Johns Hopkins (US)	Mass. General (US)	Northwestern (US)	Ottawa (Canada)	Penn State (US)	UAB (US)
Enrolled, No.	15	12	12	9	10	13	9	16	10	9
Clinical Diagnosis, No. (%)										
PD	5	5	6	5	5	5	3	5	5	5
MSA	6	2	3	1	1	3	2	6	2	0
PSP	4	5	3	3	4	5	4	5	3	4
Age, mean (SD), years	67.9 (7.1)	72.5 (7.7)	65 (8.5)	64.3 (5.7)	68.8 (5.8)	69.4 (5.3)	66.4 (9.3)	68.4 (7.9)	67.9 (10.5)	68.6 (6.9)
Sex, No. (%)										
Male	13	8	8	3	7	3	5	10	7	5
Female	2	4	4	6	3	10	4	6	3	4
Time since parkinsonism diagnosis, mean (SD), years										
PD	5.2 (1.4)	6.2 (1.2)	5.0 (2.3)	6.1 (1.5)	6.9 (0.5)	6.9 (0.9)	5.4 (1.0)	6.4 (0.6)	5.2 (0.4)	5.8 (1.7)
MSA	3.1 (1.8)	2.3 (1.9)	4.3 (2.5)	0 (n/a)	2.9 (n/a)	0.7 (0.6)	1.4 (0.8)	0.7 (1.0)	2.7 (1.7)	n/a (n/a)
PSP	1.0 (0.7)	3.7 (2.8)	0.4 (2.4)	0.3 (1.9)	0.6 (0.3)	1.2 (1.2)	2.2 (2.6)	0.6 (1.3)	5.1 (0.8)	0.5 (1.0)
3 Tesla MRI vendor	GE	Siemens	GE	Siemens	Phillips	Siemens	Siemens	Siemens	Siemens	Siemens
Diffusion directions	48	64	48	64	48	30	64	30	64	30
Maximum b-value, s/mm^2	1000	1000	1000	1000	1000	1000	1000	1000	1000	1000
Resolution, mm^3	2	2	2	2	2	2	2	2	2	2
Echo time, ms	76	67	56	58	62	91	58	85	58	82
Repetition time, ms	17000	8500	8500	7500	7482	13649	7500	10100	7500	9600

© 2025 Vaillancourt DE et al. *JAMA Neurology*.

Characteristic	UCSD (US)	UCSF (US)	Chicago (US)	Florida (US)	Kentucky (US)	Michigan (US)	Minnesota (US)	South Florida (US)	Toronto (Canada)	Wake Forest (US)	Wash U. (US)
Enrolled, No.	15	14	17	20	10	17	8	13	6	10	4
Clinical Diagnosis, No. (%)											
PD	5	7	5	5	4	5	4	5	4	5	1
MSA	4	2	4	6	2	3	0	4	0	0	2
PSP	6	5	8	9	4	9	4	4	2	5	1
Age, mean (SD), years	69.5 (8.3)	64.4 (9.9)	69.8 (8.9)	67.9 (7.4)	65.9 (4.9)	69.4 (6.1)	64.8 (10.0)	67.4 (7.6)	64.2 (5.0)	67.9 (8.6)	68.0 (8.5)
Sex, No. (%)											
Male	9	10	14	9	7	10	5	8	4	6	4
Female	6	4	3	11	3	7	3	5	2	4	0
Time since parkinsonism diagnosis, mean (SD), years											
PD	6.2 (1.2)	4.6 (1.4)	5.8 (1.6)	6.7 (0.5)	5.3 (1.8)	5.7 (1.7)	7.9 (1.0)	8.3 (1.5)	5.0 (0.7)	6.2 (1.7)	8.7 (n/a)
MSA	2.5 (1.4)	7.8 (8.0)	0.2 (1.5)	1.2 (0.9)	0.8 (1.0)	0.1 (1.5)	n/a (n/a)	1.7 (1.1)	n/a (n/a)	n/a (n/a)	4.5 (3.9)
PSP	4.0 (2.4)	1.7 (1.1)	1.0 (1.0)	1.7 (1.5)	0.8 (1.2)	1.0 (0.9)	1.8 (2.4)	1.5 (2.7)	1.1 (0.2)	0.4 (1.2)	0.3 (n/a)
3 Tesla MRI vendor	GE	GE	Phillips	Siemens	Siemens	Phillips	Siemens	GE	GE	Siemens	Siemens
Diffusion directions	48	48	32	64	64	32	64	32	32	48	64
Maximum b-value, s/mm^2	1000	1000	1000	1000	1000	1000	1000	1000	1000	1000	1000
Resolution, mm^3	2	2	2	2	2	2	2	2	2	2	2
Echo time, ms	61	55	88	58	61	83	58	82	61	82	58
Repetition time, ms	7800	9022	10095	7500	8100	12820	7500	17000	8050	9600	7500

Full site names in order of appearance: Albany Medical College, Augusta University, Beth Israel Deaconess Medical Center, Duke University Medical Center; Johns Hopkins University, Massachusetts General Hospital, Northwestern University, Ottawa Hospital Research Institute, Pennsylvania State University, University of Alabama of Birmingham, University of California San Diego, University of California San Francisco, University of Chicago, University of Florida, University of Kentucky, University of Michigan, University of Minnesota, University of South Florida, University of Toronto Center for Addiction and Mental Health, Wake Forest University, Washington University in St. Louis.

© 2025 Vaillancourt DE et al. *JAMA Neurology*.

eTable 2. Clinical scales for the prospective cohort.

	Total (N=249)			Train (N=104)			Test (N=145)		
**Clinical Scale, mean (SD)**	**PD**	**MSAp**	**PSP**	**PD**	**MSAp**	**PSP**	**PD**	**MSAp**	**PSP**
UPDRS, part I	1.8 (1.8)	2.6 (2.1)	3.3 (2.2)	1.6 (1.4)	3.2 (2.3)	3.3 (2.5)	2.0 (2.0)	2.1 (1.7)	3.2 (1.9)
UPDRS, part II	8.5 (4.6)	21.2 (8.2)	21.7 (7.5)	7.2 (4.6)	20.4 (9.2)	22.3 (7.6)	9.4 (4.4)	22.1 (7.3)	21.3 (7.5)
UPDRS, part III	22.0 (10.3)	40.1 (15.3)	39.8 (14.9)	20.8 (11.9)	38.5 (14.8)	40.2 (14.7)	22.8 (9.1)	41.6 (16.0)	39.5 (15.1)
UPDRS, total	32.4 (13.6)	64.0 (22.6)	64.8 (21.4)	29.6 (15.6)	62.1 (23.0)	65.8 (21.7)	34.2 (12.0)	65.7 (22.5)	64.1 (21.4)
Epworth, total	7.1 (4.6)	7.7 (4.6)	7.3 (5.6)	6.3 (4.5)	7.9 (4.9)	7.8 (4.8)	7.7 (4.6)	7.4 (4.4)	6.9 (6.0)
Hamilton, total	6.7 (5.9)	12.3 (6.7)	10.4 (6.8)	7.2 (6.3)	13.2 (6.5)	11.0 (7.4)	6.4 (5.6)	11.4 (6.9)	10.0 (6.4)
MoCA, total	26.4 (2.7)	23.6 (5.8)	20.1 (6.9)	26.6 (2.7)	24.1 (4.0)	19.5 (8.0)	26.3 (2.7)	23.1 (7.1)	20.6 (6.2)
Schwab and England, total	89.8 (7.7)	56.0 (27.9)	52.0 (26.9)	89.5 (8.3)	65.0 (24.4)	54.7 (26.4)	90.0 (7.3)	47.3 (28.6)	50.1 (27.4)
PDQ-39, total	21.5 (16.9)	63.5 (24.2)	59.4 (24.2)	21.2 (20.2)	60.0 (26.0)	59.9 (23.9)	21.8 (14.6)	66.8 (22.3)	59.0 (24.7)
PSP-RS, total	10.5 (5.0)	28.5 (12.4)	40.4 (13.7)	9.7 (5.2)	26.9 (12.4)	40.3 (14.0)	10.9 (4.8)	30.1 (12.4)	40.5 (13.5)
UMSARS, part I	7.8 (4.0)	23.5 (8.2)	21.1 (7.3)	6.9 (4.1)	21.5 (8.0)	21.4 (7.2)	8.4 (3.9)	25.4 (7.9)	20.8 (7.4)
UMSARS, part II	11.3 (4.6)	23.4 (9.2)	23.6 (8.6)	10.4 (4.9)	21.3 (8.5)	23.6 (8.9)	11.9 (4.3)	25.4 (9.4)	23.7 (8.4)
UMSARS, total	19.1 (7.4)	46.9 (16.8)	44.7 (14.8)	17.3 (7.8)	42.8 (15.9)	45.0 (14.7)	20.4 (6.9)	50.9 (16.9)	44.5 (14.9)

Abbreviations: Epworth: Epworth Sleepiness Scale; Hamilton: Hamilton Depression Rating Scale; MoCA: Montral Cognitive Assessment; PSP-RS: Progressive Supranuclear Palsy Rating Scale; PDQ-39: Parkinson's Disease Questionnaire; Schwab and England: Schwab and England Activities of Daily Living Scale; UMSARS: Unified Multiple System Atrophy Rating Scale; UPDRS: Unified Parkinson's Disease Rating Scale.

© 2025 Vaillancourt DE et al. *JAMA Neurology*.

eFigure 1. AIDP retest of primary endpoint model.

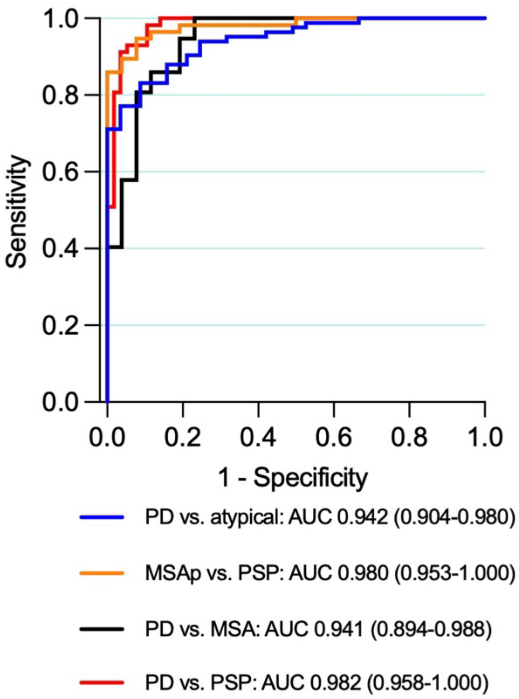

Testing set ROC-AUC curves for the retest of the primary model endpoints (Table 2, Figure 3 of the main document): PD versus atypical parkinsonism (blue), MSA versus PSP (orange), PD versus MSA (black), and PD versus PSP (red). The model included diffusion FW and FAt, age, and sex as input features. FW and FAt input features for the retest model were obtained from a second and consecutively acquired 3T diffusion scan during the clinical visit. The AUC and 95% confidence interval are reported for each endpoint.

© 2025 Vaillancourt DE et al. *JAMA Neurology*.

eFigure 2. AIDP primary and secondary endpoints for the diffusion only model.

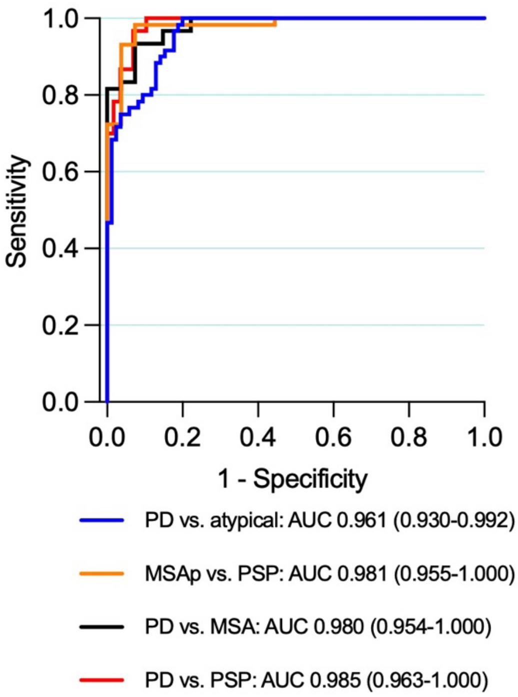

Testing set ROC-AUC curves for the primary model endpoints (Table 2, Figure 3 of the main document) without the inclusion of age and sex as input features (i.e., diffusion MRI features only): PD versus atypical parkinsonism (blue), MSA versus PSP (orange), PD versus MSA (black), and PD versus PSP (red). The AUC and 95% confidence interval are reported for each endpoint.

© 2025 Vaillancourt DE et al. *JAMA Neurology*.

eFigure 3. Regression analyses for diagnosis duration and symptom severity scores with atypical probability estimates.

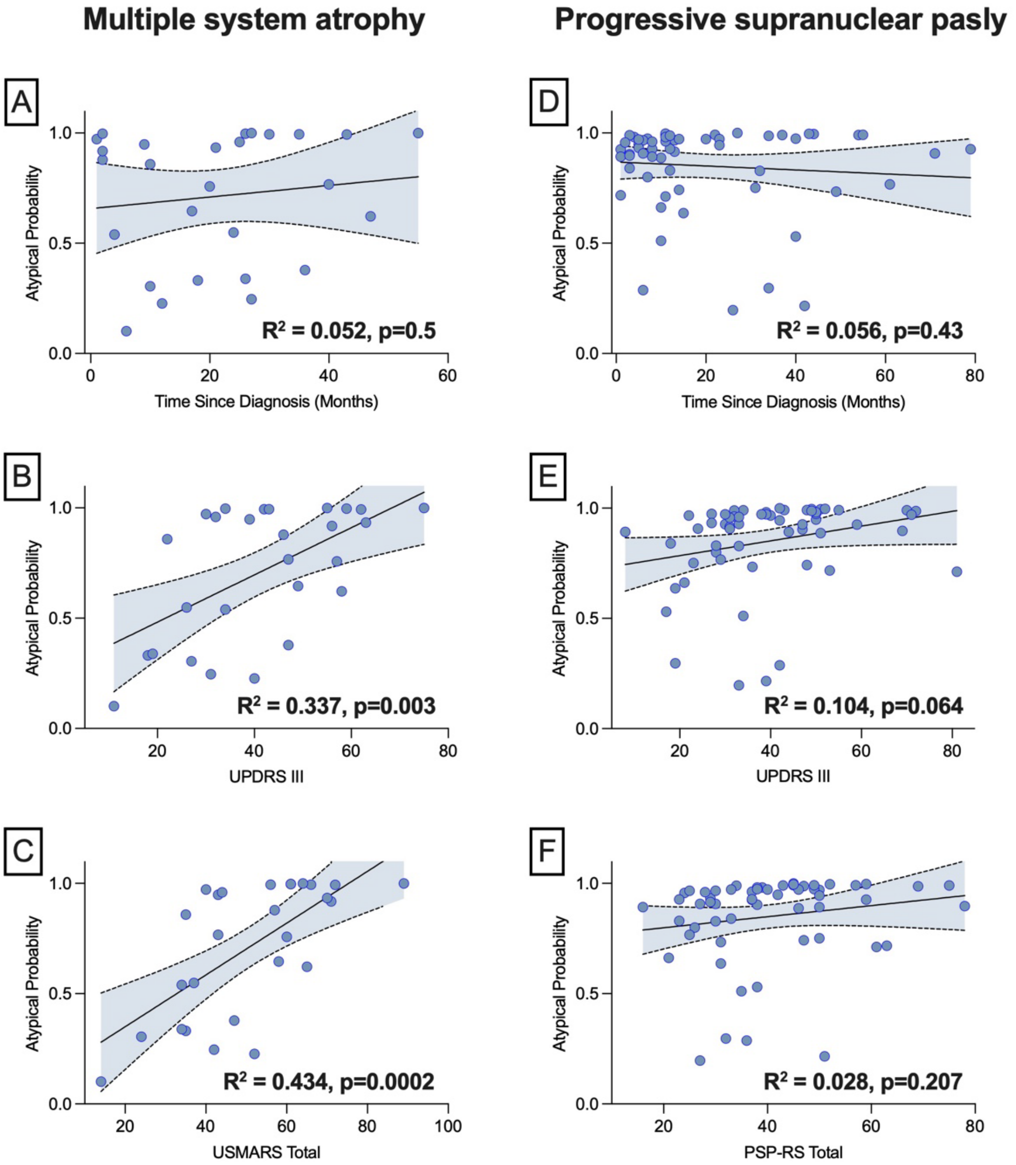

The left panel shows the regression plots for predicted atypical probability estimates (y-axis) in MSA patients by (A) diagnosis duration in months, (B) motor symptom severity as assessed by the Unified PD Rating Scale, part III (UPDRS III), and (C) MSA symptom severity as assessed by the Unified MSA Rating Scale (x-axes). The right panel shows the regression plots for predicted atypical probability estimates (y-axis) in PSP patients by (D) diagnosis duration in months, (E) motor symptom severity as assessed by UPDRS III, and (C) PSP symptom severity as assessed by the PSP Rating Scale (PSP-RS). The probability estimates were obtained from the testing set of the primary endpoint model for PD versus atypical parkinsonism. A probability estimate >0.5 indicates that the classification is weighted toward atypical parkinsonism, whereas a probability estimate <0.5 indicates that the classification is weighted toward PD. Shaded areas represent the 95% confidence interval on the regression estimate.

© 2025 Vaillancourt DE et al. *JAMA Neurology*.

eFigure 4. AIDP site preservation model for primary endpoints.

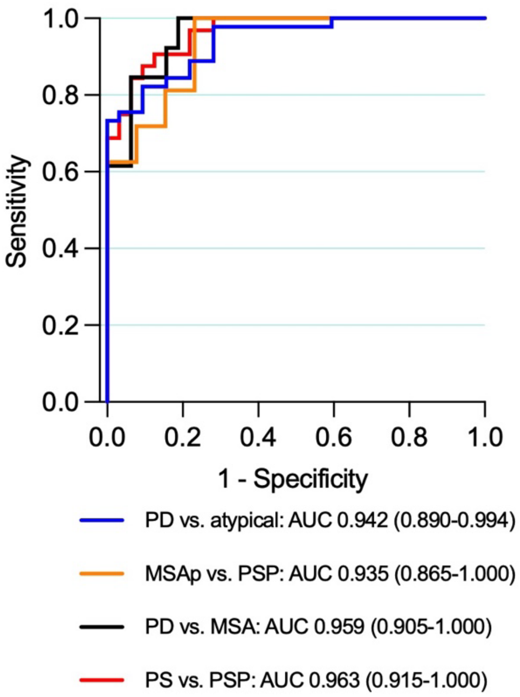

Testing set ROC-AUC curves for primary endpoints in the site preservation model: PD versus atypical parkinsonism (blue), MSA versus PSP (orange), PD versus MSA (black), and PD versus PSP (red). Patients from six of the 21 sites were exclusively held out for independent testing of the model. The AUC and 95% confidence interval are reported for each endpoint.

© 2025 Vaillancourt DE et al. *JAMA Neurology*.

eFigure 5. AIDP neuropathology model endpoints.

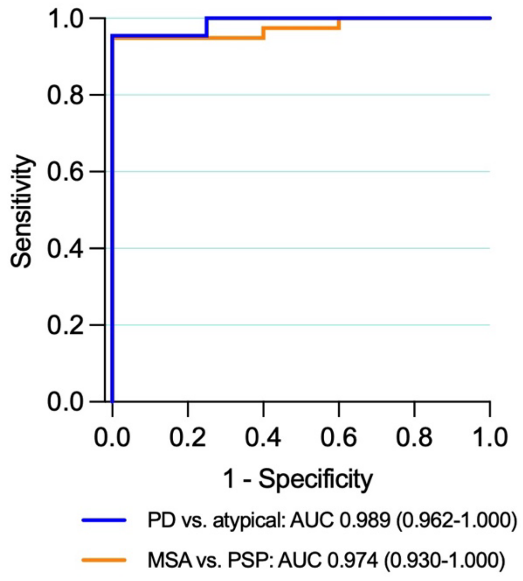

Testing set ROC-AUC curves for PD versus atypical parkinsonism (blue) and MSA versus PSP (orange) in the neuropathology validation model. The AUC and 95% confidence interval are reported for each endpoint.

© 2025 Vaillancourt DE et al. *JAMA Neurology*.

eFigure 6. Use of AIDP in a diagnostic patient workup.

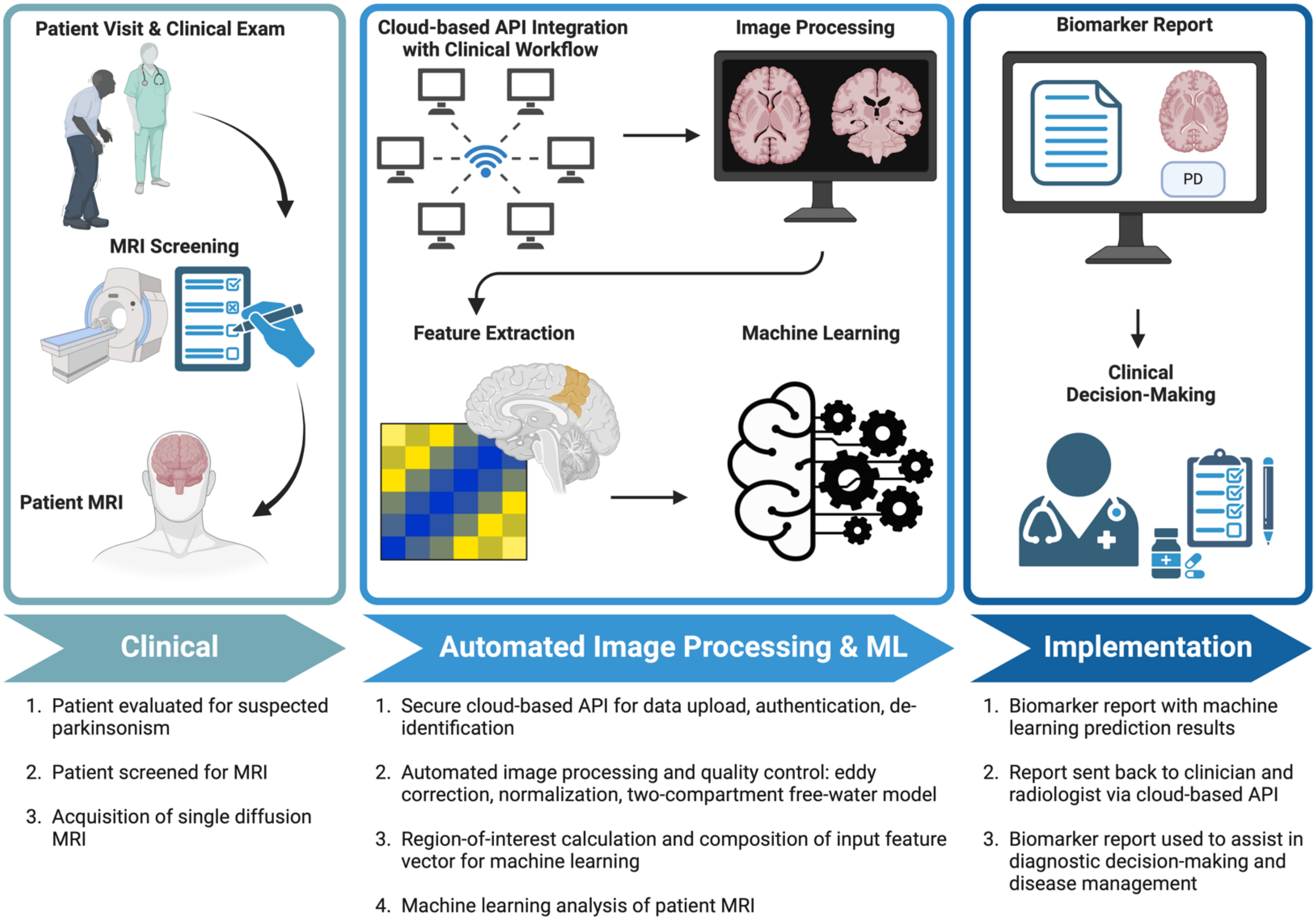

Automated Imaging Differentiation for Parkinsonism (AIDP) is a machine learning software that assists neurologists, radiologists, and other physicians differentiate between PD, MSA parkinsonian variant, and PSP. AIDP is designed for cloud-based integration with picture archiving and communication systems and medical image viewers, providing streamlined reporting of imaging findings during clinical workflows. An application programming interface (API) allows users to authenticate, upload data, and retrieve results across platforms. The user-provided input is a single-shell diffusion-weighted MRI scan acquired using widely available 3 Tesla scanners from major vendors (Siemens, General Electric, Philips). The software receives the image in DICOM format using a secure data uploading and encrypted transmission process. The software analyzes the image using machine learning and provides a report to assist in clinical diagnostic decision-making. Figure created with BioRender.com.

© 2025 Vaillancourt DE et al. *JAMA Neurology*.

eReferences

1. Hughes, A. J., Daniel, S. E., Kilford, L. & Lees, A. J. Accuracy of clinical diagnosis of idiopathic Parkinson's disease: a clinico-pathological study of 100 cases. *Journal of Neurology, Neurosurgery & Psychiatry* **55**, 181–184 (1992).
2. Hughes, A. J., Daniel, S. E., Ben-Shlomo, Y. & Lees, A. J. The accuracy of diagnosis of parkinsonian syndromes in a specialist movement disorder service. *Brain* **125**, 861–870 (2002).
3. Höglinger, G. U. *et al.* Clinical diagnosis of progressive supranuclear palsy: The movement disorder society criteria: MDS Clinical Diagnostic Criteria for PSP. *Mov Disord.* **32**, 853–864 (2017).
4. Gilman, S. *et al.* Second consensus statement on the diagnosis of multiple system atrophy. *Neurology* **71**, 670–676 (2008).
5. Archer, D. B. *et al.* Development and validation of the automated imaging differentiation in parkinsonism (AID-P): a multicentre machine learning study. *Lancet Digit Health* **1**, e222–e231 (2019).
6. Archer, D. B. *et al.* Magnetic Resonance Imaging and Neurofilament Light in the Differentiation of Parkinsonism. *Movement Disorders* **35**, 1388–1395 (2020).
7. Arpin, D. J. *et al.* Diffusion Magnetic Resonance Imaging Detects Progression in Parkinson's Disease: A Placebo-Controlled Trial of Rasagiline. *Mov Disord* **37**, 325–333 (2022).
8. Pasternak, O., Sochen, N., Gur, Y., Intrator, N. & Assaf, Y. Free water elimination and mapping from diffusion MRI. *Magn Reson Med* **62**, 717–730 (2009).
9. Wang, Y. *et al.* Quantification of increased cellularity during inflammatory demyelination. *Brain* **134**, 3590–3601 (2011).

© 2025 Vaillancourt DE et al. *JAMA Neurology*.
