# Supplementary material for: Automated Imaging Differentiation for Parkinsonism
Source: JAMA Neurol. 2025 Mar 17;82(5):495–505. doi: 10.1001/jamaneurol.2025.0112 (PMC11915115; doi:10.1001/jamaneurol.2025.0112)
Supplement: Supplement 2. — Data Sharing Statement. [file jamaneurol-e250112-s002.pdf]

Data Sharing Statement

Vaillancourt. Automated Imaging Differentiation for Parkinsonism. *JAMA Neurol*. Published March 17, 2025. doi:10.1001/jamaneurol.2025.0112

Data
Data available: Yes
Data types: Deidentified participant data
How to access data: Method determined by corresponding author.
When available: With publication

Supporting Documents
Document types: None

Additional Information
Who can access the data: researchers whose proposed use of the data has been approved by corresponding author and study team
Types of analyses: any purpose approved by corresponding author and study team
Mechanisms of data availability: to be determined at the time of request
